# Chinese herbal medicine formulas as adjuvant therapy for osteonecrosis of the femoral head: A systematic review and meta-analysis of randomized controlled trials

**DOI:** 10.1097/MD.0000000000012196

**Published:** 2018-09-07

**Authors:** Qingwen Zhang, Fan Yang, Yaolong Chen, Haibin Wang, Delong Chen, Wei He, Peng Chen

**Affiliations:** a First Affiliated Hospital; b First School of Clinical Medicine, Guangzhou University of Chinese Medicine, Guangzhou, Guangdong; c Evidence-Based Medicine Center, School of Basic Medical Sciences, Lanzhou University, Lanzhou, Gansu, China.

**Keywords:** adjuvant therapy, Chinese herbal medicine formulas, meta-analysis, osteonecrosis of the femoral head, PRISMA—driven systematic review

## Abstract

**Background:**

Osteonecrosis of femoral head (ONFH) is a disabling clinical entity affecting mainly young adults. Although Chinese Herbal Medicine (CHM) has been widely used as an adjunct therapy for ONFH in China, its effectiveness is not well defined. The purpose of this systematic review is to assess the effectiveness of CHM as an adjunct therapy to core decompression (CD) for patients with ONFH.

**Methods:**

A systematic literature search was conducted in 10 electronic databases. Randomized controlled trials involving CHM and CD for ONFH were included. Two authors independently assessed the studies for inclusion and extracted the data. A meta-analysis was conducted to estimate the safety and efficacy of CHM as an adjunct therapy.

**Results:**

Twenty-three randomized controlled trials with 1815 participants were included. The formulas used in these studies were different and we only combined data of studies observing the same formulas. Patients treated with CHM additionally exhibited a better TER (total effective rate) compared with CD alone in different degree, with the risk ratio (RR) varies from 1.09 to 1.09. The Harris score and radiographic effective rate indicate a similar result, with the medicine (MD) varies from 17.35 to 14.94 and RR 1.40 to 1.27. The risk of side-effect was barely reported except only 2 study record that no complications were observed.

**Conclusion:**

This systematic review indicated that CHM as adjunctive therapy may improve the effectiveness of CD. However, a firm conclusion could not be reached because of overall high risk of bias in most domains. Further studies of higher quality are required, and other benefits of CHM remain to be determined.

## Abstract 2

Supplemental Digital Content is available in the text

## 1 Introduction

Osteonecrosis of the femoral head (ONFH) is recalcitrant disease caused by an inadequate blood supply to the affected segment of the subchondral bone.^[1]^ Annually, 20,000 to 30,000 new cases are diagnosed in the USA.^[2,3]^ In China, with its massive population, 150,000 to 200,000 new cases of ONFH are diagnosed every year.^[4]^ Because a significant number of patients with ONFH are young (20–40 years old), and the long-term survivorship of prosthesis is uncertain, preserving their own joints is preferable. Encouraging results of hip joint preservation at short-term and midterm follow-up have been reported.^[5–7]^ Core decompression (CD), one of the classic joint-sparing approaches, results in excellent outcomes, particularly in the precollapse stages.^[8]^ Furthermore, CD combined with other adjuvant treatment methods has demonstrated positive results.^[9]^

Complementary medicine and alternative medicine (CAM) are commonly used among ONFH patients because of the absence of specific agents. The types of CAM in patients with ONFH vary across countries. In China, Chinese herbal medicine (CHM) has been used for several 100 years to treat ONFH (named bone erosion in Huang Di Nei Jing). CHM is used in nearly all ONFH patients with Chinese backgrounds.^[10,11]^ According to the World Health Organization report in 2001, CHM accounted for 30% to 50% of total medical consumption in mainland China.^[12]^ CHM has been widely used either for disease treatment or health maintenance. Accumulating evidence suggests that CHM combined with CD have been used to treat ONFH by removing the necrotic tissue and promoting blood circulation. Therefore, the efficiency of CHM as 1 adjuvant treatment for ONFH should be comprehensively assessed.

To our knowledge, no systematic review has assessed the effectiveness of CHM as an adjunctive therapy for ONFH. Our study aimed to evaluate the curative effects and side effects about the use of CHM with CD in treating ONFH.

## 2 Method

Our systematic review of the literature is performed following the Cochrane Handbook for Systematic Reviews of Interventions and reported according to the PRISMA guidelines. The secondary research was made in our study by reviewing previous studies without involving any human or animal research subjects, thus, ethical review is not required for our study.

### 2.1 Eligibility criteria

1. Types of studies: Randomized control trials were considered. The language of the reports was restricted to English and Chinese. No publication date or publication status restrictions were imposed.
2. Types of participants: Participants diagnosed with ONFH based on clinical history or radiographic changes were considered,^[13]^ and patients should not have received similar conservative operations on the involved hip previously. The stage of ONFH was restricted to Stage I or II according to ARCO (Association Research Circulation Osseous) classification or the Ficat and Alert classification method^[14]^ for the reason of high risk of deterioration and unsatisfied curative effect in stage III and IV patients. Studies that included a majority of juveniles were excluded from this review.
3. Types of intervention: Trials compared the effectiveness of CD combined with CHM formula and CD alone as control intervention. The core decompression procedure comprises center decompression and multiple drilling for core decompression with or without cancellous bone impaction grafting. Studies were excluded if differences in types of CD procedure between treatment group and control group occurred. Trials were also excluded if the participants received other types of treatments (pharmacotherapy, physical therapy, etc.) that simultaneously proved to be effective for ONFH.
4. Types of outcome measurements: The outcomes of included trials should have contained at least 1 of the 3 types of measurements: clinical measurements (Harris scores), radiography measurements (AP or frog-leg lateral radiographs, computed tomography or magnetic resonance imaging), and effectiveness evaluation.

### 2.2 Database search

Published papers were searched in various databases, including the Cochrane Library, MEDLINE, Embase, ISI Web of Knowledge, the Chinese Biomedical Literature Database, Wanfang Database, China National Knowledge Infrastructure, Weipu database and Japanese Institutional Repositories Online using the following primary search items: Femur Head Necrosis, Chinese traditional medicine, herbal decoctions, and core decompression. **(**S1 Appendix, S2 Appendix**).** The final search was conducted on October 18, 2017.

In addition, potential proceedings, books, ongoing studies, and conference papers were searched and evaluated. We searched references from the included studies for any possible titles matching the inclusion criteria.

### 2.3 Study selection and data extraction

After screening titles and abstracts based on the search strategy, we included all potential articles that met our inclusion criteria. The full text of expected inclusions was carefully examined. Remaining studies that could potentially be included in our analysis were reviewed by 2 independent reviewers.

Two reviewers extracted data independently based on a predefined data-extraction form that included characteristics of participants (including sample size, duration of follow-up, age, gender, stage and severity of ONFH, etiology); details of intervention (including formulas use, type of CD, dosage, duration and frequency of the formula, postoperative management); and type of outcome measurement (including Harris hip score, radiographic evaluation, total efficiency).

### 2.4 Outcome assessment

We regarded the following as outcome measures:

Primary outcome: The majority of studies divided total efficiency into 4 levels following the Curative Effect Standard (CES): Cure: no pain, unlimited activities, comfortable daily activities; Excellent: pain relief, scarcely limited activities, light daily activities; Effectiveness: mild pain relief and a slight improvement in daily activities; Failure: no improvement.^[15]^ The total effective rate (TER) refers to a percentage related to outcomes on the number of cures, excellent ratings and effectiveness ratings.

Secondary outcome: The Harris hip score (HHS) was used to assess clinical effectiveness based on pain level, joint function, and mobility, with a total score of 100 points.^[16]^

According to AP or frog-leg lateral radiographs, we classified the radiographic effectiveness into 3 levels: Excellent: the femoral head showed stable morphology or collapse was smaller than 2 mm, the cystic area was reduced or disappeared, and the osteopetrosis area became blurred, with or without osteoarthritis; Effectiveness: collapse of the femoral head was smaller than 4 mm, the cystic area was mildly reduced, and joint displayed slight degeneration; Failure: the collapse of the femoral head was larger than 4 mm, with significant osteoarthritis. We define the radiographic effective rate (RER) as the percentage of hips evaluated as Excellent or Effective. Variations in the necrotic area were also analyzed if related data were offered.

Herbs are types of plants, some of which have toxic effects such as cytotoxins, digitalism, and alkaloids. The adverse effects of synthetic drugs as opposed to single drugs must be considered because adverse events of most herbal drugs are relatively less frequent when used singly.^[17,18]^

### 2.5 Overall high risk of bias in most domains

To assess the quality and risk of study bias, we used the assessment tool recommended by the Cochrane Collaboration.^[19]^ This tool for assessing risk of bias includes 6 domains: random sequence generation, allocation concealment, blinding methods, incomplete outcome data, selective outcome reporting, and other sources of bias. The quality was categorized as low, unclear, or high risk for bias and risk of bias graph would summarize the results.

### 2.6 Data analysis and synthesis

For the studies using the same formula for CHM treatment, we used Cochrane Collaboration software (Review Manager Version 5.3 for Windows, *Copenhagen: The Nordic Cochrane Centre*) to perform the statistical analysis. Mean differences (MD) with a 95% confidence interval (CI) were calculated for continuous data (HHS, decrease of necrotic volume) and risk ratio (RR) with 95% CI to summarize dichotomous outcome data (TER, RER. For other studies that included great heterogeneity in their interventions, we only qualitatively analyzed the results.

The statistical heterogeneity was presented as significant when *I*^*2*^ > 50% or *P* < .1. In the absence of significant heterogeneity, we pooled data using a fixed-effect model (*I*^*2*^ < 50%); otherwise, we used a random effects model (*I*^*2*^ > 50%).

Because of the small number of trials testing the same intervention and the same outcome, a meaningful funnel plot analysis could not be conducted; therefore, the reporting biases were not assessed.

### 2.7 “Summary of findings” tables

We created “Summary of Findings” tables for different comparisons and included the following outcomes: total effective rate, HHS, and RER. The overall qualities of the evidence were assessed using the GRADE approach and incorporated into these tables.

## 3 Results

### 3.1 Search results and trial characteristics

**Figure 1:**
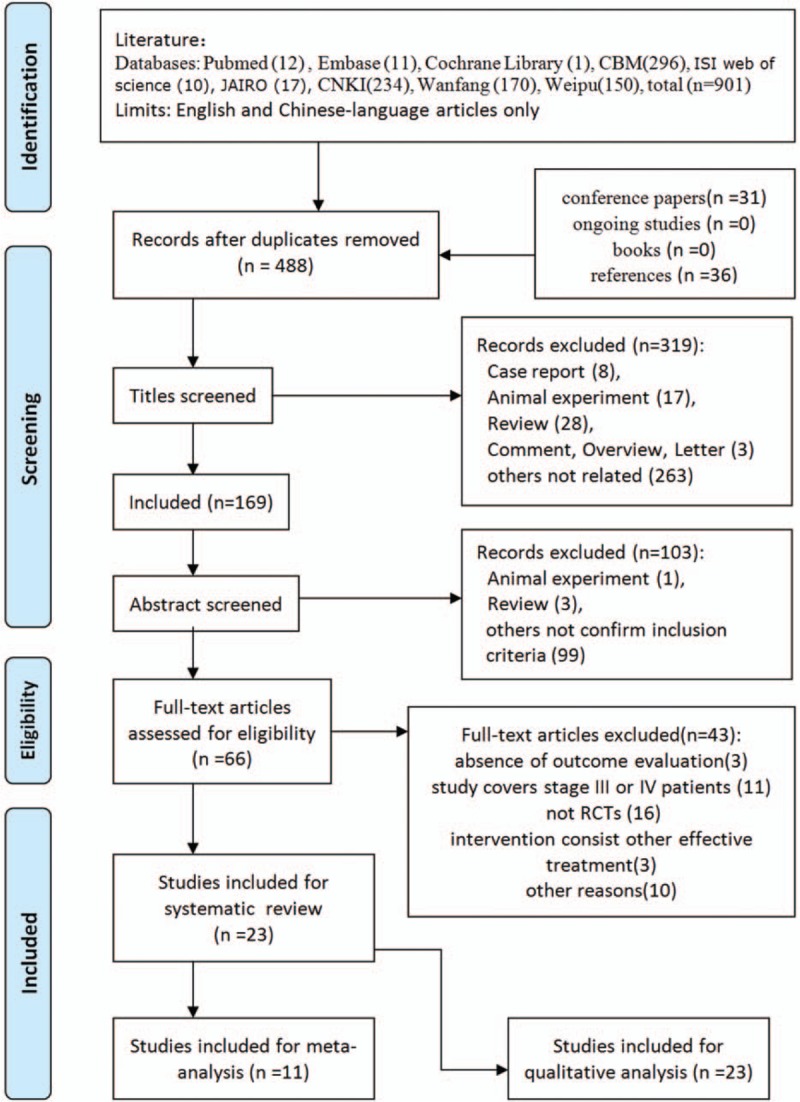
Flow diagram.

Figure 1 describes the process of study selection. The initial search identified 819 potentially relevant articles. Sixty-six potential studies merited assessment after reviewing the full text. Finally, 23 studies ^[20–42]^ met the inclusion criteria and were included in the systematic review. These involved 1815 participants, of whom 1183 (65.2%) were male and 632 (34.8%) were female. No studies that met the criteria for inclusion were identified by checking the references. All of these trials were conducted in China and published in Chinese. The primary inclusion criteria were adults (mean age across individual studies varied from 22.72–45.1) with the following etiology: trauma, steroid abuse, alcohol abuse, and idiopathy.

Among the included studies were 12 methods using TCM formulas including “Bushenhuoxue decoction (nourishing kidney and activating blood recipe),” ^[21,33,41]^ “Huoxuejiangu decoction (blood-activating and bone-invigorating recipe),” ^[20]^ “Guhuaisi No.2 decoction (The No. 2 osteonecrosis decoction),” ^[24]^ “Jianbuhuqian pill (limb-strengthening pills),”^[22]^ “Luguishenggu pill (bone-generating pills with deer horn and tortoiseshell),” ^[23]^ “Sijunzi decoction (decoction of four noble drugs), Taohongsiwu decoction (Menstro Ease Decoction),” ^[25]^ “Huoluogukang pill (collateral-activating and bone-invigorating pills),” ^[27]^ “Wentonghuoxue decoction (warm, smooth, blood-activating decoction),” ^[29]^ “Yiqihuoxuebushentongluo decoction (Qi-tonifying, blood-activating, kidney-tonifying, and collateral-dredging decoction),” ^[35]^ “Jiangu decoction (bone-invigorating decoction),” ^[40]^ “Self-drafting TCM formula,” ^[37,42]^ and “formula based on syndrome differentiation.” ^[26,28,30–32,34,36,38,39]^ The duration of treatment ranged from 2 months to 12 months, and the follow-up observation of patients ranged from 3 months to 2 years after finishing treatment to evaluate whether the intervention was effective. All included studies have mentioned the diagnostic criteria by introduce the details or cited a reference which accord with eligibility criteria. None of these reports mentioned quality control for herbs used in original studies. The detailed baseline characteristics and interventions are summarized in Table 1.

**Table 1:**
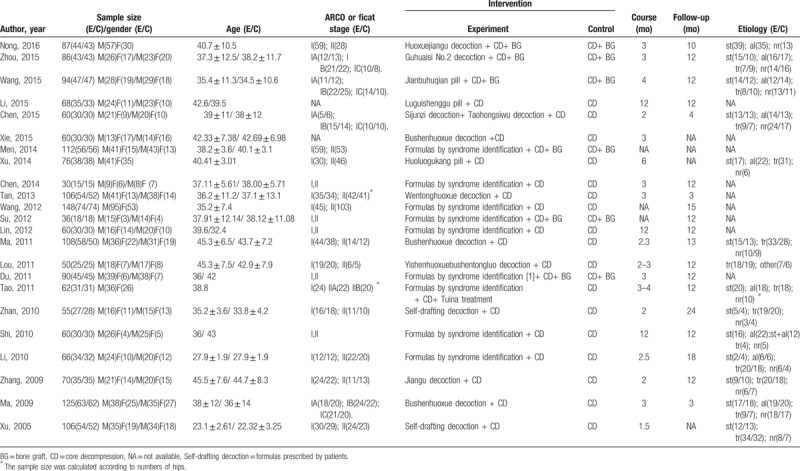
Characteristics of included studies.

### 3.2 Methodological quality of included trials

**Figure 2:**
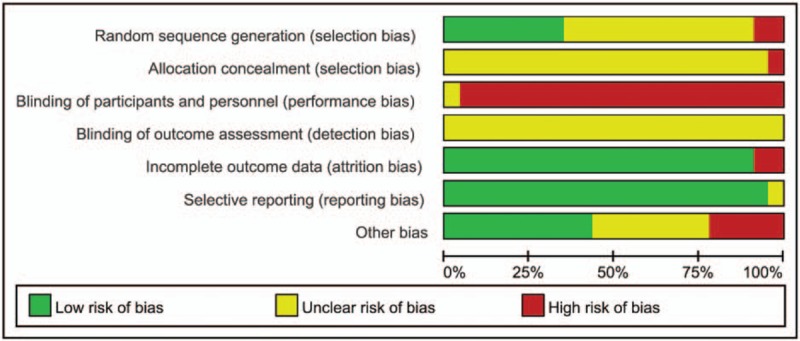
Risk of bias graph.

**Figure 3:**
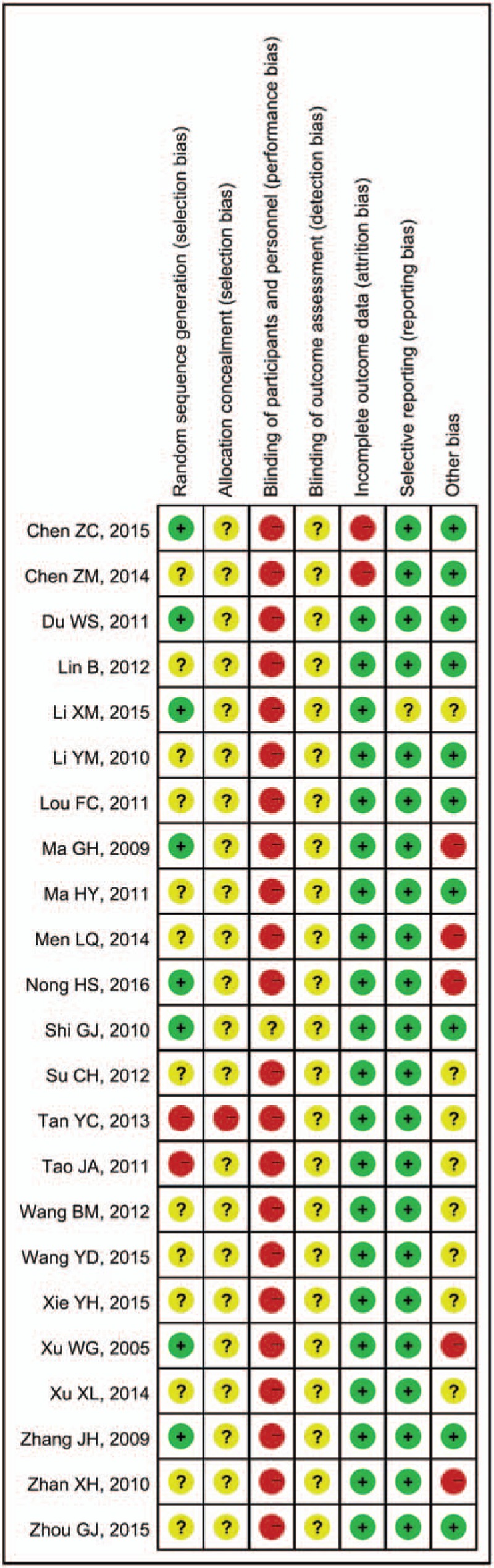
Risk of bias summary.

Figures 2 and 3 present the risk of bias graph and summary, respectively.

The randomized allocation of participants was mentioned in all of the included trials, and 8 trials^[20,23,25,36,38,40–42]^ presented the methods for sequence generation, including the use of random number tables. Two trials ^[29,34]^ described a semirandom method in the sequence generation process that was generated by order of hospitalization. However, insufficient information was provided to judge whether the trial was conducted properly. Allocation concealment was not mentioned in any of the trials. None of the trials used the blinding of participants and personnel because no trials used a placebo in the control group; the blinding of outcome assessment was barely mentioned in these studies. No study reported treatment withdrawals, dropout, or loss to follow-up because of lack of efficacy. We have searched these studies in Chinese Clinical Trial Register. But we found no record about these studies and all included studies reported no trial registration. According to the results reported by each study, no data were missing and the data described in the Methods section were reported in the Results section after the final follow-up. This reporting may suggested complete outcome data and no selective reporting, but potential risk of bias may be caused.

### 3.3 Effect of the interventions

Because heterogeneity existed in the formulas used in the majority of the excluded studies, we only synthesized data extracted from studies researching the same formula or similar herbs as the adjunct intervention. The follow-up times differed slightly across these studies, and some reports did not report the duration of follow-up. However, this lack would not induce obvious heterogeneity because of the relatively long-term course of ONFH, and all of the studies included could be classified as short-term effectiveness observations. We analyzed the data from the remaining studies qualitatively according to risk bias and characteristics. The outcomes of included studies are described in Table 2.

**Table 2:**
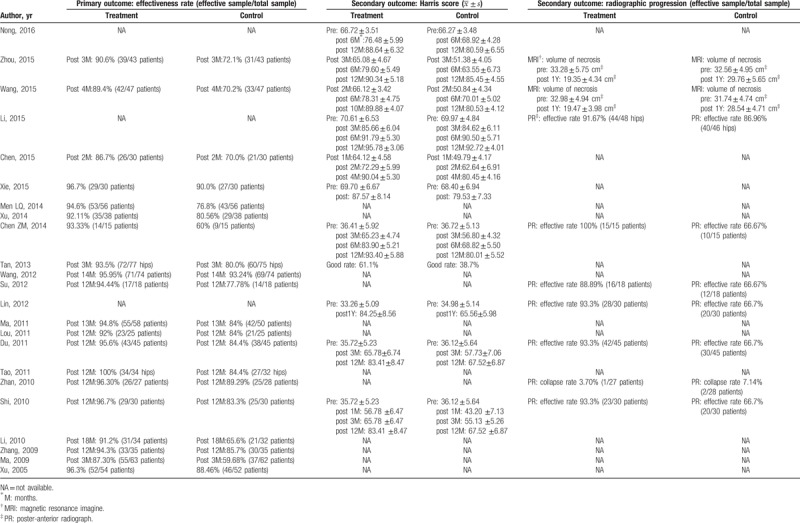
The outcome of included studies.

### 3.4 Formulas by syndrome identification with CD (FSI group) compared with CD alone

Nine studies selected formulas by syndrome identification as the assistant intervention. For the reason of the diversity of syndrome, there may exist several protocol of choosing decoctions. And 4 of these studies ^[26,28,30,36]^ observed the efficiency of using the same method **(**Protocol 01, S3 Appendix **).**

**Figure 4:**
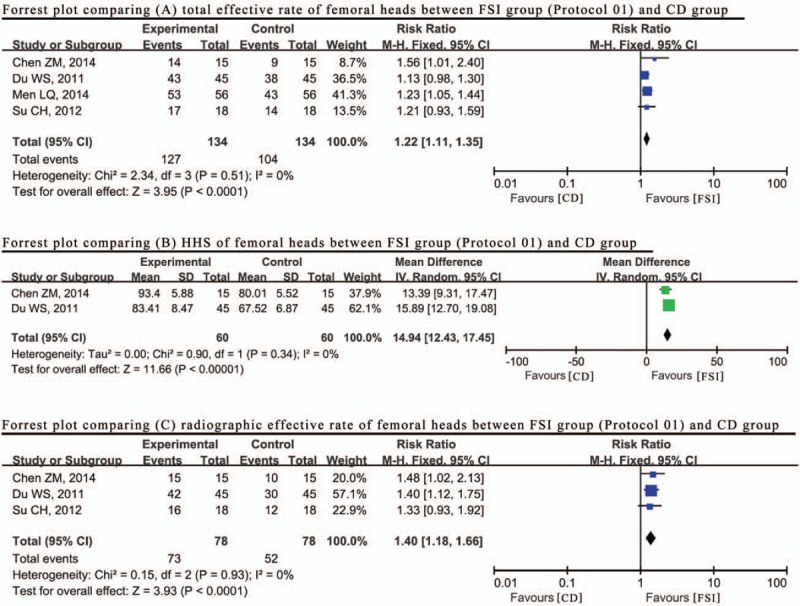
Forrest plot comparing results (A) total effective rate, (B) HHS, (C) radiographic effective rate between FSI (formulas by syndrome identification) group (Protocol 01) and CD (core decompression) group. HHS = Harris hip score.

Figure 4 indicates that no heterogeneities were observed between these 2 groups (*P*=.51, I^2^=0%). Thus, the fixed effect model was used to combine the number of TER of the FSI group compared with the CD group. The overall meta-analysis indicated that the RR was 1.22 (95% CI: 1.11–1.35), suggesting that the treatment of CD combined with formulas by syndrome identification obtained a relatively high total effective rate compared with CD alone. Among the 4 studies above, this result was consistent with the outcome of HHS evaluation (MD=14.94; 95% CI: 12.43–17.45; *P*=.34, I^2^ = 0%) and the radiographic effective rate (RR = 1.40; 95% CI:1.18–1.66; *P* = .93, I^2^ = 0%) based on a synthesis of 2 studies ^[26,36]^ that reported HHS results and 3 studies ^[26,30,36]^ that reported the radiographic effective rate (Fig. 4).

**Figure 5:**
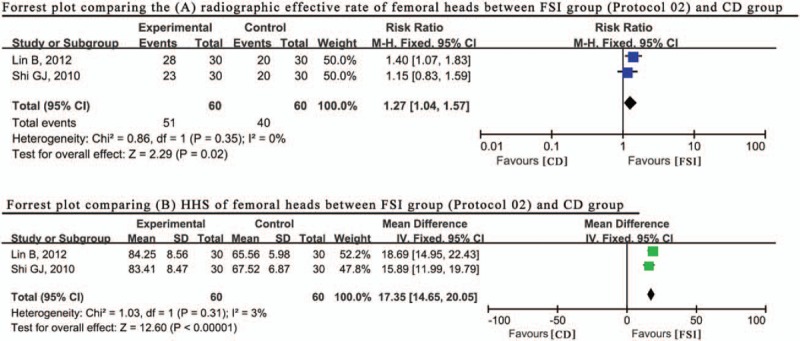
Forrest plot comparing results (A) total effective rate, (B) HHS between FSI (formulas by syndrome identification) group (Protocol 02) and CD (core decompression) group. HHS = Harris hip score.

Another method of using formulas by syndrome identification was used by 2 other studies (Protocol 02).^[32,38]^Figure 5 indicates that there was no obvious heterogeneity between the results of the radiographic effective rate across these 2 studies (*P* = .31, I^2^ = 3%); Therefore, the fixed effect model was used to combine data abstracted from those studies. The results of this data synthesis indicate that this method combined with CD also attains a better radiographic performance than CD alone (RR = 1.27, 95% CI: 1.04–1.57). In addition, the HHS results of these 2 studies suggested that this method combined with CD also obtained better results in clinical evaluation (MD = 17.35, 95% CI: 14.65–20.05, *P* = .31, I^2^ = 3%), verifying the conclusion above (Fig. 5).

Of the remaining 3 studies that investigated formulas by syndrome identification, Wang et al^[31]^ reported on CD combined with their own method of composing formulas, obtaining a higher total effective rate than the CD group 14 months after intervention. Li et al^[39]^ arrived at an identical conclusion after 18 months of follow-up. Tuina therapy (massage therapy) was additionally used after surgery in an observation group in a study conducted by Tao et al.^[34]^ After 12 months of follow-up, the observation group obtained a total effective rate of 100%, compared with 84.4% in the control group. However, because Tuina therapy was not used in the control group and the quasi-randomized method of this study, there was great potential risk of bias in this study.

### 3.5 Bushenhuoxue decoction with CD compared with CD alone

**Figure 6:**
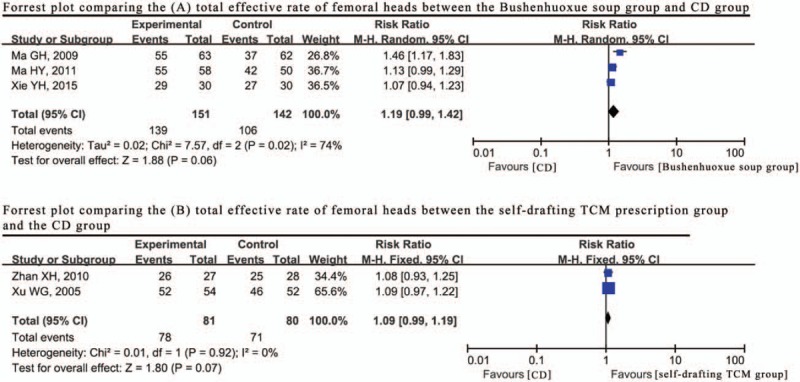
Forrest plot comparing results (A) total effective rate, (B) total effective rate between other therapeutic method and CD (core decompression) only.

The use of Bushenhuoxue decoction with CD was observed in 3 trials.^[21,33,41]^ For the reason of some different herbs used in this formula between these 3 studies, there exist a significant heterogeneity among studies (*P* = .02, I^2^ = 74%) (Fig. 6), the random effects model was used to combine the data of the total effective rate, and the RR was 1.19 (95% CI: 0.99–1.42). Xie et al^[21]^ further reported the HHS results, suggesting that Bushenhuoxue decoction improved the results of the clinic evaluation following CD.

### 3.6 Self-drafting traditional Chinese medical formula with CD compared with CD alone

Two studies^[37,42]^ used the same self-drafting formula as the adjuvant therapy with CD, and their course of formula treatment differed slightly, indicating a small heterogeneity (*P* = .92, I^2^ = 0%) (Fig. 6). Therefore, the data regarding the total effective rate was synthesized using a fixed effect model and arrived at the RR of 1.09 (95% CI: 0.99–1.19), indicating that this self-drafting formula could improve the therapeutic effects of CD.

### 3.7 Other formula with CD compared with CD alone

The remaining 9 studies used various formulas as the adjuvant therapy, and we qualitatively analyzed the results from the following 3 perspectives: total effective rate, radiographic evaluation, and HHS result.

Xu et al^[27]^ used the Huoluogukang pill as the adjunctive treatment to CD. Compared with CD treatment alone, this formula increased the total effective rate (92.11% compared with 77.32%). Yiqihuoxuebushentongluo decoction and Jiangu decoction were examined by studies conducted by Lou et al^[35]^ and Zhang et al^[40]^ respectively. The total effective rates that those authors identified support the conclusion that the formula combined with CD obtained a better curative effect than CD alone.

Tan et al^[29]^ divided groups according to order of admission and researched the effectiveness of Wentonghuoxue decoction combined with CD on the basis of HHS results and total effective rate. The HHS improved significantly, and the respectable (HSS>80) rate of the group that used Wentonghuoxue decoction was obviously higher than the control group (61.1% compared with 38.7%). The total effective rate after 3 months was also consistent with this conclusion.

Li et al^[23]^ evaluated the effectiveness of the Guilushenggu pill as an adjunctive treatment for CD. The HHS and radiographic effective rate, dynamically reported at 3, 6, and 12 months following surgery, resulted in a higher HHS in the observation group than in the control group; however, the difference was relatively mild. Regarding the radiographic appearance, patients who took Guilushenggu pills after CD obtained a higher radiographic effective rate, indicating a better efficiency than CD only.

In addition to the outcomes evaluation above, the survival rate of the hip joint was further reported by a study conducted by Nong et al.^[20]^ Those authors used Huoxuejiangu decoction as the adjunctive therapy of CD and observed no significant difference between the 2 groups. Nevertheless, the HHS and radiographic effective rate suggested better results.

In a study conducted by Zhou, magnetic resonance imaging were performed on participants as the radiographic evaluation, and the volume of necrosis was calculated.^[24]^ Compared with the control group, participants using No.2 osteonecrosis decoction experienced a more obvious decrease in the necrotic area. The results of HHS and the total effective rate (90.6% compared with 72.1%) also verified the efficiency of the decoction's use after CD. This conclusion was reached by Wang et al ^[22]^ by the same method used to evaluate the results of the Jianbuhuqian pill.

Chen et al^[25]^ observed the effectiveness of Taohongsiwu decoction combined with Sijunzi decoction as the adjunct therapy with CD, resulting in a higher HHS and total effective rate in the observation group than in the control group (86.7% compared with 70.0%). The hemorheology test also indicated a greater improvement in participants who accepted prescriptive therapy.

### 3.8 Adverse event

Only 2 studies ^[27,37]^ reported no complications following CD treatment. Related complications, such as infection or hip joint ankyloses, were not reported in other studies; however, we could not verify from the evidence that the rate of complications was zero.

### 3.9 Summary of findings

S4–7 Appendixes summarize the quantitative analysis above and the overall quality of the evidence by outcome using the GRADE approach.

According to the summary above, the meta-analysis and qualitative analysis both indicated that CHM could improve the clinical outcomes of ONFH patients as well as the radiographic evaluation with rare risk of side-effect.

## 4 Discussion

This study was the first systematic review that showed a trend of herbal medicine improving quality of life in patients with ONFH. The systematic review was also the first time synthesis results were presented for each formula. Wang's systematic review on osteoporosis presented the results of the different components of diseases ^[20]^ and included 12 RCTs, which are different from the trials we included. Although Chen's systematic review included 10 RCTs of Chinese herbal medicine treatments for ONFH, the results were reported based on comparing 5 interventions in controlled groups, which is also different from our study because Chen included only one controlled intervention.^[44]^ Ye's meta-analysis indicated that CHMs promote blood circulation and decrease blood stasis; however, there were no detailed formulas in the review.^[45]^

The Cochrane methodology strengthens our review. In fact, some components in these herbs were effective, such as nutritional bone drugs in clinical treatment. A longer period of observation and a larger sample size may be required to test the effects scientifically. Furthermore, the evidence that the treatment decreased adverse effects or mortality or the outcome of the “ZHENG” report was insufficient.

With regard to clinical symptoms, ONFH always falls into the category of “bone erosion. ” Generally, 1 formula comprises 4 categories to achieve a common effect: ministerial herbs, deputy herbs, assistant herbs, and envoy herbs. In TCM theory, “blood stasis” (“Xue Yu” in Chinese) plays a crucial role in the pathological mechanism of osteonecrosis, which results from decreased blood circulation. All formulas included in our review promote blood circulation and decrease stasis. It may be necessary to use the active ingredient(s) from ministerial herbs as an illustration for further mechanistic study in promoting new tissue regeneration.^[43,46,47]^

*Psoralea corylifolia* L (PCL, Buguzhi in Chinese) is a widely used medicinal plant. Bioactive ingredients extracted from PCL are used to treat bone fractures, osteomalacia, and osteoporosis. Wang et al reported that bavachin significantly stimulated osteoblast proliferation, indicating its ability to promote bone formation. Bavachin also protects blood cells against oxidative hemolysis, thereby indirectly preventing blood stasis.^[48]^*Angelica Sinensis* (AS, Danggui in Chinese) is useful in wound healing, promoting collagen secretion, angiogenesis and granulation tissue formation, which play a role in “removing the necrosis for tissue regeneration.”^[49,50]^ The aqueous extract from AS may also directly enhance bone formation and protein secretion in a dose-dependent manner in vitro. *Rehmannia glutinosa* Libosch (RGL, Dihuang in Chinese) has been widely used for treatments relating to blood and the immune and endocrine systems. Extract of RGL in a 2-herb formula plays a vital role in promoting improvement in these areas.^[51]^ Ethanol extraction of RGL has preventive effects on bone loss by stimulating the proliferation and activities of osteoblasts while inhibiting the generation and resorptive activities of osteoclasts in vivo and in vitro. *Ligusticum wallichii* (LW, ChuanXiong in Chinese) has been used for rheumatic arthralgia and coronary heart diseases. Ferulic acid (FA) and ligustilide, as the primary bioactive ingredients of CX, are used to improve blood fluidity and inhibit platelet aggregation, exhibiting strong antioxidant activity.^[52,53]^ These formulas and ingredients are advantageous in bone tissue regeneration and angiogenesis, particularly in treating diseases related to “bone erosion.”

CD by drilling is merely a surgical technique for relieving intraosseous pressure in the femoral head. An anastomosis induced by drilling between the circulatory systems of bone was demonstrated and the importance of the periosteum confirmed in an animal study.^[54]^ Furthermore, the duration of the decreased core pressure induced by drilling is too short for substitution of a necrotic area and could explain the inferior clinical results of the procedure. Thus, a combination of herbal medicine and CD has been a good choice for treating early stages of ONFH.

There are several limitations in this review. First, although randomization was mentioned in all studies, only 3 of them described the randomization procedure in the random number table with very limited information. We also tried to contact those authors to confirm the RCT but did not receive any reply. Therefore, we believed that some of the claimed RCTs were not the real ones. Second, for the outcome reporting, all but one of the included studies reported no adverse events associated with the herbs. Certainly, herbal drugs or CD repair are accompanied by the possibility of adverse events. Although it is widely accepted that herbal medicines are safe to use for various diseases in China, the formulations, dosages, and manufacturing processes were prepared by the investigators without a reasonably detailed rationale, and the quality management methods for their tested interventions are unknown. Thus, it is necessary for safety requirements to be reported appropriately because there has been limited and inadequate reporting of adverse events. Finally, publication bias and other biases may exist. Although we conducted comprehensive searches and tried to avoid language and location bias, we could not exclude potential publication bias because all included studies were published in China. The ease of retrieving literature, related papers and scientific results and reviews in many countries other than China, however, is quite limited. Such publications are difficult to identify using many academic databases in those countries, possibly because of great uncertainty and the reliance on former experiences with herbal medicine and failure to provide sufficient emphasis.

## 5 Conclusions

In conclusion, the reported effectiveness and safety of Chinese herbal medicine in combination with core decompression for ONFH can be taken as encouraging but not convincing. Because the included studies were poorly designed and low quality, the evidence remains inconclusive. Further RCTs with adequate allocation concealment, the blinding of participants and assessors, or sample size estimation will be required to effectively evaluate the effectiveness of this combination of therapies. Any adverse events and the effectiveness of long-term follow-up will also be reported.

## Acknowledgment

The authors thank Guibo Xing, PhD (Senior Statistician at University of California, Davis, CA) for helpful suggestions on the statistics analysis and copyediting.

## Author contributions

**Conceptualization:** Qingwen Zhang, Fan Yang.

**Data curation:** Yaolong Chen, Haibin Wang.

**Methodology:** Qingwen Zhang.

**Software:** Delong Chen.

**Writing – original draft:** Qingwen Zhang, Fan Yang, Yaolong Chen, Peng Chen.

**Writing – review & editing:** Wei He, Peng Chen.

## Supplementary Material

Supplemental Digital Content

## References

[1] Malizos KN Karantanas AH Varitimidis SE Osteonecrosis of the femoral head: etiology, imaging and treatment. Eur J Radiol 2007;63:16–28. 17555906 10.1016/j.ejrad.2007.03.019

[2] Lavernia CJ Sierra RJ Grieco FR Osteonecrosis of the femoral head. J Am Acad Orthop Surg 1999;7:250–61. 10434079 10.5435/00124635-199907000-00005

[3] Petrigliano FA Lieberman JR Osteonecrosis of the hip: novel approaches to evaluation and treatment. Clin Orthop Relat Res 2007;465:53–62. 17906590 10.1097/BLO.0b013e3181591c92

[4] Yin JM Zhao L Zhao SC Relationship between the Apolipoprotein AI, B gene polymorphism and the risk of non-traumatic osteonecrosis. Lipids Health Dis 2014;13:149. 25248404 10.1186/1476-511X-13-149 PMC4247152

[5] Song WS Yoo JJ Kim Y-M Results of multiple drilling compared with those of conventional methods of core decompression. Clin Orthop Relat Res 2007;454:139–46. 16906081 10.1097/01.blo.0000229342.96103.73

[6] Israelite C Nelson CL Ziarani CF Bilateral core decompression for osteonecrosis of the femoral head. Clin Orthop Relat Res 2005;441:285–90. 16331016 10.1097/01.blo.0000192365.58958.84

[7] Marker DR Seyler TM Ulrich SD Do modern techniques improve core decompression outcomes for hip osteonecrosis? Clin Orthop Relat Res 2008;466:1093–103. 18392909 10.1007/s11999-008-0184-9 PMC2311489

[8] Kerimaa P Väänänen M Ojala R MRI-guidance in percutaneous core decompression of osteonecrosis of the femoral head. Eur Radiol 2016;26:1180–5. 26228899 10.1007/s00330-015-3905-y

[9] Pierce TP Jauregui JJ Elmallah RK A current review of core decompression in the treatment of osteonecrosis of the femoral head. Curr Rev Musculoskelet Med 2015;8:228–32. 26045085 10.1007/s12178-015-9280-0 PMC4596206

[10] Zhao DW Hu YC Chinese experts’ consensus on the diagnosis and treatment of osteonecrosis of the femoral head in adults. Orthop Surg 2012;4:125–30. 22927145 10.1111/j.1757-7861.2012.00192.x PMC6583607

[11] Joint Surgery Group of the Orthopaedic Branch of the Chinese Medical Association. Guideline for Diagnostic and Treatment of Osteonecrosis of the Femoral Head. Orthop Surg 2015;7:200–7. 26311093 10.1111/os.12193 PMC6583727

[12] World Health Organization. Programme on Traditional Medicine. (2001). Legal status of traditional medicine and complementary/alternative medicine: a worldwide review. Geneva: World Health Organization. http://www.who.int/iris/handle/10665/42452

[13] Mont MA Cherian JJ Sierra RJ Nontraumatic osteonecrosis of the femoral head: where do we stand today? A ten-year update. J Bone Joint Surg Am 2015;97:1604–27. 26446969 10.2106/JBJS.O.00071

[14] Mont MA Marulanda GA Jones LC Systematic analysis of classification systems for osteonecrosis of the femoral head. J Bone Joint Surg Am 2006;88:16–26. 17079363 10.2106/JBJS.F.00457

[15] Chinese state administration of traditional Chinese medicine. SAo. Chinese disease diagnosis and efficacy standards. Nanjing University Press 1994;193:200–1.

[16] Siddique T Sah RK Masood F Improvement in Harris Hip Score after cementless total hip arthroplasty in young active adults with secondary hip arthritis- A short-term follow-up result. J Pak Med Assoc 2015;65:S63–6. 26878539

[17] D’arcy PF Adverse reactions and interactions with herbal medicines. Part 2—Drug interactions. Adverse Drug React Toxicol Rev 1993;12:147–62. 8218717

[18] De Smet PA Health risks of herbal remedies. Drug Saf 1995;13:81–93. 7576267 10.2165/00002018-199513020-00003

[19] Higgins JP, Green S. *Cochrane Handbook for Systematic Reviews of Interventions.* Vol 5. Wiley Online Library; 2008.

[20] Nong HS Blood-Activating and bone-invigorating recipe as assistant therapy of core decompression combined with impacting bone graft: a therapy study for non-traumatic femur head necrosis before collapse. Mod J Integr Traditional Chin Western Med 2016;25:1456–8.

[21] Xie JH Li S Nourishing kidney and activating blood recipe combined with multihole core decompression for early stage avascular necrosis of femoral head: a clinical observation of 30 cases. Hunan J Traditional Chin Med 2015;31:81–3.

[22] Wang YD Liu Y Wang JX A curative effect analysis of Jianbuhuqian pill as assistant therapy of core decompression combined with bone graft for femur head necrosis in early stage. Guiding J Traditional Chin Med Pharm 2015;21:59–61.

[23] Li XM Guo DH Shi GJ A short-term curative effect analysis of Guilushenggu pill combined with core decompression for femur head necrosis in early stage. Chin J Surg Integr Traditional Western Med 2015;21:173–5.

[24] Zhou GJ Core decompression combined with the No.2 osteonecrosis decoction as a treatment of femur head necrosis in early stage: a clinical study of 43 cases. Guiding J Traditional Chin Med Pharm 2015;21:82–4.

[25] Chen ZC Li B Li JZ The clinical efficacy of Taohongsiwu decoction combined with Sijunzi decoction as an adjuvant therapy for femur head necrosis in early stage: a clinical study of 30 cases. Guiding J Traditional Chin Med Pharm 2015;21:85–7.

[26] Chen ZM Syndrome differentiation of TCM combined with core decompression and bone grafting, as a treatment of femur head necrosis in early stage, a clinical study. China J Chin Med 2014;29:1751–5.

[27] Xu XL Clinical efficacy of treating femoral head avascular necrosis with the Huoluo Gukang pill plus pith decompression. Clin J Chin Med 2014;6:106–7.

[28] Men LQ Hu R A clinical analysis of treating 112 cases of early femoral head necrosis in TCM medicine differentiation. Clin J Chin Med 2014;6:91–2.

[29] Tan YC Li CS A curative effect analysis of Wentonghuoxue decoction combined with core decompression for femur head necrosis in early stage. J Bethune Military Med College 2013;11:230–1.

[30] Su CH Syndrome differentiation of TCM combined with core decompression and bone grafting for femur head necrosis in early stage, a observation study. Asia-Pacific Traditional Med 2012;8:57–8.

[31] Wang BM The conbined treatment of traditional chinese medicine and western medicine for avascular necrosis of femoral head: a clinical curative effect analysis. China Foreign Med Treat 2012;22:117–9.

[32] Lin B The clinical curative effect analysis of early stage necrosis of femoral head. Chin Foreign Med Re 2012;10:35–6.

[33] Ma HY Mou CL Zhan JX Nourishing kidney and activating blood recipe combined with core decompression for early stage avascular necrosis of femoral head in adults: a clinical observation study. Mod J Integr Traditional Chin Western Med 2011;20:1988–9.

[34] Tao JA The treatment of femur head necrosis: syndrome differentiation of TCM combined with core decompression. Chin J Ethnomedicine Ethnopharmacy 2011;16:89–93.

[35] Lou FC Traditional Chinese medicine combined with core decompression as a treatment for femur head necrosis in early and middle stage. Jilin Med J 2011;32:2783–4.

[36] Du WS Han FW Xie YL Syndrome differentiation of Chinese herb combined with core decompression and bone grafting for femur head necrosis in early stage, a study of 45 cases. Chin J Exp Traditional Med Formulae 2011;17:271–2.

[37] Zhan XH The conbined treatment of traditional Chinese medicine and western medicine for femur head necrosis in stage I-II, a clinical observation of 27 cases. Guiding J Traditional Chin Med Pharm 2010;16:54–5.

[38] Shi GJ Liu SJ Zhang JL Core decompression combined with traditional Chinese medicine as a treatment for femur head necrosis in early stage, a clinical study of 45 cases. J Yunnan Univ Traditional Chin Med 2010;33:42–6.

[39] Li YM Zhou WF Zou YM The effect of conbined treatment of taditional Chinese medicine and westem medicine in patients with early avascular necrosis of the femoral head. Chin Foreign Health Abstract 2010;7:275–7.

[40] Zhang JH The conbined treatment of traditional Chinese medicine and western medicine for femur head necrosis, a clinical observation of 35 cases. Guiding J Traditional Chin Med Pharm 2009;15:51–2.

[41] Ma GH A clinical observation study of nourishing kidney and activating blood recipe combined with core decompression for early stage avascular necrosis of femoral head. J Emerg Traditional Chin Med 2009;18:547–8.

[42] Xu WG Lin Y Clinical observation on the treatment of 54 cases of aseptic necrosis of femoral head: the integration TCM with Western medicine. Guiding J TCM 2005;11:52–4.

[43] Wang ZQ Li JL Sun YL Chinese herbal medicine for osteoporosis: a systematic review of randomized controlled trails. Evid Based Complement Alternat Med 2013;2013:356260. 23431336 10.1155/2013/356260 PMC3572690

[44] Chen LL Wei HE Zhang QW Systematic review on Chinese medicine for osteonecrosis of the femoral head. China J Traditional Chin Med Pharm 2012;27:710–5.

[45] Ye JH Niu Y Meta Analysis of Femoral Head Necrosis Treated by TCM Intervention Therapy of Promoting Blood Circulation and Removing Blood Stasis. J Guangxi College Traditional Chin Med Univ 2002;5:71–3.

[46] Li WF Jiang JG Chen J Chinese medicine and its modernization demands. Arch Med Res 2008;39:246–51. 18164973 10.1016/j.arcmed.2007.09.011

[47] Pan SY Chen SB Dong HG New perspectives on Chinese herbal medicine (Zhong-Yao) research and development. Evid Based Complement Alternat Med 2011;2011:403709. 21785622 10.1093/ecam/neq056 PMC3135515

[48] Wang D Li F Jiang Z Osteoblastic proliferation stimulating activity of Psoralea corylifolia extracts and two of its flavonoids. Planta Med 2001;67:748–9. 11731919 10.1055/s-2001-18343

[49] Lim SH Ha TY Kim SR Ethanol extract of Psoralea corylifolia L. and its main constituent, bakuchiol, reduce bone loss in ovariectomised Sprague–Dawley rats. Br J Nutr 2009;101:1031–9. 18801207 10.1017/S0007114508066750

[50] Hsiao CY Hung CY Tsai TH A study of the wound healing mechanism of a traditional Chinese medicine, Angelica sinensis, using a proteomic approach. Evid Based Complement Alternat Med 2012;2012:467531. 22536285 10.1155/2012/467531 PMC3319019

[51] Liu CL Tam JC Sanders AJ Molecular angiogenic events of a two-herb wound healing formula involving MAPK and Akt signaling pathways in human vascular endothelial cells. Wound Repair Regen 2013;21:579–87. 23755905 10.1111/wrr.12055

[52] Oh KO Kim SW Kim JY Effect of Rehmannia glutinosa Libosch extracts on bone metabolism. Clin Chim Acta 2003;334:185–95. 12867291 10.1016/s0009-8981(03)00238-9

[53] Wang J Yuan Z Zhao H Ferulic acid promotes endothelial cells proliferation through up-regulating cyclin D1 and VEGF. J Ethnopharmacol 2011;137:992–7. 21782921 10.1016/j.jep.2011.07.019

[54] Simank H-G Graf J Kerber A Long-term effects of core decompression by drilling. Cells Tissues Organs 1997;158:185–91. 9394955

