# Supplementary material for: Chinese herbal medicine formulas as adjuvant therapy for osteonecrosis of the femoral head: A systematic review and meta-analysis of randomized controlled trials
Source: Medicine (Baltimore). 2018 Sep 7;97(36):e12196. doi: 10.1097/MD.0000000000012196 (PMC6133442; doi:10.1097/MD.0000000000012196)
Supplement: Supplemental Digital Content [file medi-97-e12196-s001.docx]

**Chinese Herbal Medicine Formulas as Adjuvant Therapy for Osteonecrosis of the Femoral Head: A PRISMA -Driven Systematic Review**

Qingwen Zhang^1¶^, Fan Yang^2¶^, Yaolong Chen^3&^, Haibin Wang^1&^,Delong Chen^2^, Wei He^1*^, Peng Chen^1*^

**S1 Appendix. Search strategy: pubmed**

| Search | Query |
| --- | --- |
| #21 | #7 AND #16 AND #20 |
| #20 | #16 OR #17 OR #18 |
| #19 | Bouche decompression |
| #18 | center decompression |
| #17 | core decompression |
| #16 | #8 OR #9 OR #10 OR #11 OR #12 OR #13 OR #14 OR #15 |
| #15 | herbal decoctions |
| #14 | formula |
| #13 | herbal medicine |
| #12 | traditional medicine treatment |
| #11 | TCM |
| #10 | Chinese prescription |
| #9 | Traditional medicine treatment |
| #8 | Medicine, Chinese Traditional[MeSH Terms] |
| #7 | #1 OR #2 OR #3 OR #4 OR #5 OR #6 |
| #6 | Femoral Head Avascular Necrosis |
| #5 | Femoral Head Aseptic Necrosis |
| #4 | Femoral Head Necrosis |
| #3 | Femoral Head Ischemic Necrosis |
| #2 | Femur Head Necrosis |
| #1 | Femur Head Necrosis[MeSH Terms] |

**S2 Appendix. Search strategy: Chinese database**

| Search | Query |
| --- | --- |
| #21 | #4 AND #16 AND #20 |
| #20 | #17 OR #18 OR #19 |
| #19 | 钻孔减压 |
| #18 | 髓心减压 |
| #17 | 髓芯减压 |
| #16 | #5 OR #6 OR #7 OR #8 OR #9 OR #10 OR #11 OR #12 OR #13 OR #14 OR #15 |
| #15 | 传统医学 |
| #14 | 传统治疗 |
| #13 | 中西结合 |
| #12 | 中医 |
| #11 | 中西医 |
| #10 | 汤剂 |
| #9 | 方剂 |
| #8 | 方药 |
| #7 | 草药 |
| #6 | 中药 |
| #5 | 中草药[MeSH Terms] |
| #4 | #1 OR #2 OR #3 |
| #3 | 股骨头缺血性坏死 |
| #2 | 股骨头无菌性坏死 |
| #1 | 股骨头坏死[MeSH Terms] |

**S3 Appendix. The prescriptions used by included studies**

| 2014/Men | Dialectical medication (Protocol 01) | deficiency of liver and kidney: **Huqian pill**;  phlegm-dampness blocking: **Zhimifuling pill;**  deficiency of Qi and blood: **Huangqiguizhiwuwu soup;**  Qi-stagnation and blood stasis: **Shentongzhuyu soup;**  wind-cold-wetness bype of arthralgia: **Duhuojisheng soup** |
| --- | --- | --- |
| 2014/Chen | Dialectical medication  (Protocol 01) | deficiency of liver and kidney: **Huqian pill**;  phlegm-dampness blocking: **Zhimifuling pill;**  deficiency of Qi and blood: **Huangqiguizhiwuwu soup;**  Qi-stagnation and blood stasis: **Shentongzhuyu soup;**  wind-cold-wetness bype of arthralgia: **Duhuojisheng soup** |
| 2012/Su | Dialectical medication  (Protocol 01) | deficiency of liver and kidney: **Huqian pill**;  phlegm-dampness blocking: **Zhimifuling pill;**  deficiency of Qi and blood: **Huangqiguizhiwuwu soup;**  Qi-stagnation and blood stasis: **Shentongzhuyu soup;**  wind-cold-wetness bype of arthralgia: **Duhuojisheng soup** |
| 2011/Du | Dialectical medication  (Protocol 01) | deficiency of liver and kidney: **Huqian pill**;  phlegm-dampness blocking: **Zhimifuling pill;**  deficiency of Qi and blood: **Huangqiguizhiwuwu soup;**  Qi-stagnation and blood stasis: **Shentongzhuyu soup;**  wind-cold-wetness bype of arthralgia: **Duhuojisheng soup** |
| 2012/Lin | Dialectical medication  (Protocol 02) | 1 month after operation: **Qili powder**;  2-3 months after operation：**Xuguhuoxue soup;**  4-12 months after operation: **Zhuangyaojianshen soup** |
| 2010/Shi | Dialectical medication  (Protocol 02) | 1 month after operation: **Qili powder;**  2-3 months after operation：**Xuguhuoxue soup;**  4-12 months after operation: **Zhuangyaojianshen soup** |
| 2012/Wang | Dialectical medication | **deficiency of liver and kidney:** Astragalus 40g、ginger 30g, herba epimedii 25g,  tortoise shell 20g, turtle shell 20g, earthworm 20g, medlar 20g, dogwood 20g,  atractylodes 15g, colla corii asini 15g, dragon's blood 5g  **vital energy and blood stasis:** rhizoma corydalis 50g, radix achyranthis bidentatae 30g, safflower carthamus 30g, woodlouse 25g, Caulis Spatholobi 25g, Ligusticum wallichii 25g, asarum 20g, peach kernel 20g, red paeonia 20g, frankincense 15g,  Manis pentadactyla 15g, myrrh 15g, radix curcumae 15g  **qi-blood deficiency:** Astragalus 50g, Codonopsis pilosula 40g, fructus psoraleae 30g, teasel root 30g, medlar 25g, Angelica sinensis 25g, Stephania tetrandra 20g, cortex moutan radicis 20g, Eucommia ulmoides 20g |
| 2010/Li | Dialectical medication | **vital energy and blood stasis:** Taohongsiwu soup;  **deficiency of liver and kidney:** Liuweidihuang pill;  **qi-blood deficiency:** Shengxuebusui soup |
| 2011/Tao | Dialectical medication | **vital energy and blood stasis:** Angelica sinensis 12g, Ligusticum wallichii 9g,  red paeonia 9g, Salvia miltiorrhiza 12g, Manis pentadactyla 9g, earthworm 9g,  radix clematidis 12g, radix curcumae 9g, Sappanwood 6g, rehmannia 12g, woodlouse 6g, liquorice 3g  **qi deficiency and blood stasis:** Astragalus 30g, Codonopsis pilosula 15g, Salvia miltiorrhiza 12g, Manis pentadactyla 9g, red paeonia 9g, earthworm 9g, rehmannia 11g, Angelica sinensis 9g, Ligusticum wallichii 9g, radix clematidis 12g, Caulis Spatholobi 15g, liquorice 3g  **phlegm-dampness blocking**: Codonopsis pilosula 12g, atractylodes rhizome 12g, Poria cocos 15g, dendrobe 9g, semen coicis 30g, peach kernel 9g, Pinellia ternata 9g, pericarpium citri reticulatae 9g, semen brassicae 9g, pawpaw 15g, Achyranthes bidentata 9g, radix clematidis 12g, liquorice 3g  **deficiency of liver and kidney:**  Astragalus 30g, Angelica sinensis 12g， rehmannia 12g， medlar 20g, dogwood 20g, teasel root 30g, cortex cinnamomi 6g, bidentate achyranthes 9g, dragon's blood 4g |
| 2015/Xie | Bushenhuoxue decoction | rehmannia 15g, Eucommia ulmoides 10g, teasel root 10g, medlar 10g, fructus psoraleae 10g, Semen Cuscutae 10g, Angelica sinensis 10g, frankincense 5g, myrrh 5g, radix angelicae pubescentis 10g, fructus evodiae 5g, flowers carthami 10g, pseudo-ginseng 10g, cistanche 10g |
| 2011/Ma | Bushenhuoxue decoction | Angelica sinensis 12g, Ligusticum wallichii 9g, rehmannia 20g, bidentate achyranthes 15g, pseudo-ginseng 3g, leech 9g, atractylodes rhizome 15g, Chinese yam 15g, bark of peony tree 12g, rhizoma cibotii 15g, herba epimedii 12g, Schisandra chinensis 9g, cistanche 20g |
| 2009/Ma | Bushenhuoxue decoction | Rehmanni, dogwood, Eucommia ulmoide, Rhizoma Drynariae, teasel root, parasitic loranthus, tortoise plastron, bidentate achyranthes, red-rooted salvia, Ligusticum wallichii |
| 2010/Zhan | Self-drafting TCM prescription | rehmannia 20g, medlar 15g, Chinese yam 20g, Eucommia ulmoides 10g, rhizoma cibotii 15g, deer-horn glue 10g, bidentate achyranthes 10g, pseudo-ginseng 10g, scorpio 6g, stiff silkworm 10g, Ligusticum wallichii 15g, Manis pentadactyla 10g |
| 2005/Xu | Self-drafting TCM prescription | rehmannia 20g, medlar 15g, Chinese yam 20g, Eucommia ulmoides 10g, rhizoma cibotii 15g, deer-horn glue 10g, bidentate achyranthes 10g, pseudo-ginseng 10g, scorpio 6g, stiff silkworm 10g, Ligusticum wallichii 15g, Manis pentadactyla 10g |
| 2016/Nong | Huoxuejiangu soup | Astragalus 50 g, Ligusticum wallichii 10g, red-rooted salvia 10g, hematoxylon 10g, paeony root20g, Poria cocos 20g, Angelica sinensis 15g, pseudo-ginseng 15g,  bidentate achyranthes 15g |
| 2015/Zhou | No.2 osteonecrosis decoction | bidentate achyranthes 20g, peach kernel 10g, flowers carthami 10g, Angelica sinensis 10g, red-rooted salvia 10g, pawpaw 5g, Rhizoma Drynariae 10g, Costustoot 10g, teasel root 20g |
| 2015/Wang | Jianbuhuqian pill | rhizoma anemarrhenae 10g, golden cypress 10g, Glue of tortoise Plastron 10g, deer-horn glue 10g, rehmannia 15g, bidentate achyranthes 10g, white paeony root 10g, Eucommia ulmoides 10g, teasel root 10g, fructus psoraleae 10g, cynomorium songaricum 10g, seed of Chinese dodder 10g, Angelica sinensis 10g, ginseng 10g, notopterygium root 10g, white atractylodes rhizome 10g, Astragalus 20g |
| 2015/Li | Guilushenggu pill | deer-horn glue, tortoise plastron, Eucommia ulmoides, rehmannia, moutan bark |
| 2015/Chen | Sijunzi soup  And  Taohongsiwu soup | Codonopsis pilosula 20g, Poria cocos 10g, white atractylodes rhizome 10g, liquorice 6g, peach kernel 12g, flowers carthami 5g, Angelica sinensis 12g, rehmannia 10g, Ligusticum wallichii 12g, white paeony root 15g |
| 2014/Xu | Huoluogukang pill | Angelica sinensis, red-rooted salvia, Astragalus, Caulis Spatholobi, Ligusticum wallichii, red paeonia, Rhizoma Drynariae, woodlouse, frankincense, myrrh |
| 2013/Tan | Wentonghuoxue soup | Angelica sinensis 9g, radix angelicae 9g, radix aconiti agrestis 9g, frankincense 15g, myrrh 15g, rehmannia 15g, dogwood 10g, white paeony root 10g, pericarpium zanthoxyli 15g, native copper 6g |
| 2011/Lou | Yiqihuoxuebushentongluo soup | Astragalus 60g, Codonopsis pilosula 20g, Angelica sinensis 15g, Ligusticum wallichii 15g, frankincense 6g, myrrh 6g, flowers carthami 6g, pawpaw 15g, scorpion 6g, earthworm 10g, korean epimedium 15g, deer-horn glue 15g |
| 2009/Zhang | Jiangu soup | Rehmannia 20g, dogwood 10g, Chinese yam 15g, moutan bark 10g, Poria cocos 10g, rhizoma alismatis 10g, pseudo-ginseng 5g, Ligusticum wallichii 6g, red-rooted salvia 15g, scorpio 6g, earthworm 10g, bidentate achyranthes 15g |

**S4 Appendix**

| **Prescriptions based on syndrome differentiation with CD (PBSD group) versus CD (Protocol 01)** | | | | | |
| --- | --- | --- | --- | --- | --- |
| **Patient or population:** patients with osteonecrosis of femoral head **Settings:** inpatients **Intervention:** CD combined with Prescriptions based on syndrome differentiation (Protocol 01) **Comparison:** CD only | | | | | |
| **Outcomes** | **Illustrative comparative risks* (95% CI)** | | **Relative effect (95% CI)** | **No of Participants (studies)** | **Quality of the evidence (GRADE)** |
|  | Assumed risk | Corresponding risk |  |  |  |
|  | **CD only** | **CD combined with Prescriptions based on syndrome differentiation (Protocol 01)** |  |  |  |
| **Total effective rate** Follow-up: 12 months | **Study population** | | **RR 1.22**  (1.11 to 1.35) | 268 (4 studies) | ⊕⊕⊕⊝ **moderate**^1,2,3^ |
|  | **776 per 1000** | **947 per 1000** (861 to 1000) |  |  |  |
|  | **Moderate** | |  |  |  |
|  | **773 per 1000** | **943 per 1000** (858 to 1000) |  |  |  |
| **HHS**  Follow-up: 12 |  | The mean HHS in the intervention groups was **14.94 higher** (12.43 to 17.45 higher) |  | 120 (2 studies) | ⊕⊕⊝⊝ **low**^4,5^ |
| **Radiographic effective rate** Follow-up: 12 months | **Study population** | | **RR 1.4**  (1.18 to 1.66) | 156 (3 studies) | ⊕⊕⊝⊝ **low**^6,7^ |
|  | **667 per 1000** | **933 per 1000** (787 to 1000) |  |  |  |
|  | **Moderate** | |  |  |  |
|  | **667 per 1000** | **934 per 1000** (787 to 1000) |  |  |  |
| *The basis for the **assumed risk** (e.g. the median control group risk across studies) is provided in footnotes. The **corresponding risk** (and its 95% confidence interval) is based on the assumed risk in the comparison group and the **relative effect** of the intervention (and its 95% CI).  **CI:** Confidence interval; **RR:** Risk ratio; | | | | | |
| GRADE Working Group grades of evidence **High quality:** Further research is very unlikely to change our confidence in the estimate of effect.  **Moderate quality:** Further research is likely to have an important impact on our confidence in the estimate of effect and may change the estimate. **Low quality:** Further research is very likely to have an important impact on our confidence in the estimate of effect and is likely to change the estimate. **Very low quality:** We are very uncertain about the estimate. | | | | | |
| ^1^ three of these four studies exist high risk of bias ^2^ This outcome was evaluated according to subjective method ^3^ The total NO. of participants were 268 ^4^ All studies exsit large risk of bias ^5^ this outcome was evaluated using subjective method ^6^ All studies exist large risk of bias ^7^ the evaluation of radiography only indicate one part of curative effect | | | | | |

**S5 Appendix**

| **Prescriptions based on syndrome differentiation with CD (PBSD group) versus CD (Protocol 02)** | | | | | |
| --- | --- | --- | --- | --- | --- |
| **Patient or population:** patients with Osteonecrosis of femoral head **Settings:** inpatients **Intervention:** CD combined with prescriptions based on syndrome differentiation (Protocol 02) **Comparison:** CD only | | | | | |
| **Outcomes** | **Illustrative comparative risks* (95% CI)** | | **Relative effect (95% CI)** | **No of Participants (studies)** | **Quality of the evidence (GRADE)** |
|  | Assumed risk | Corresponding risk |  |  |  |
|  | **CD only** | **CD combined with prescriptions based on syndrome differentiation (Protocol 02)** |  |  |  |
| **Radiographic effective rate** Follow-up: 12 months | **Study population** | | **RR 1.27**  (1.04 to 1.57) | 120 (2 studies) | ⊕⊕⊝⊝ **low**^1,2^ |
|  | **667 per 1000** | **847 per 1000** (693 to 1000) |  |  |  |
|  | **Moderate** | |  |  |  |
|  | **667 per 1000** | **847 per 1000** (694 to 1000) |  |  |  |
| **HHS** Follow-up: 12 |  | The mean hhs in the intervention groups was **17.35 higher** (14.65 to 20.05 higher) |  | 120 (2 studies) | ⊕⊕⊝⊝ **low**^1,2,3^ |
| *The basis for the **assumed risk** (e.g. the median control group risk across studies) is provided in footnotes. The **corresponding risk** (and its 95% confidence interval) is based on the assumed risk in the comparison group and the **relative effect** of the intervention (and its 95% CI).  **CI:** Confidence interval; **RR:** Risk ratio; | | | | | |
| GRADE Working Group grades of evidence **High quality:** Further research is very unlikely to change our confidence in the estimate of effect.  **Moderate quality:** Further research is likely to have an important impact on our confidence in the estimate of effect and may change the estimate. **Low quality:** Further research is very likely to have an important impact on our confidence in the estimate of effect and is likely to change the estimate. **Very low quality:** We are very uncertain about the estimate. | | | | | |
| ^1^ All studies exist large risk of bias ^2^ the evaluation of radiography only indicate one part of curative effect ^3^ this outcome was evaluated using subjective method | | | | | |

**S6 Appendix**

| **Bushenhuoxue soup with CD versus CD** | | | | | |
| --- | --- | --- | --- | --- | --- |
| **Patient or population:** patients with osteonecrosis of femoral head **Settings:** inpatients **Intervention:** Bushenhuoxue soup with CD **Comparison:** CD only | | | | | |
| **Outcomes** | **Illustrative comparative risks* (95% CI)** | | **Relative effect (95% CI)** | **No of Participants (studies)** | **Quality of the evidence (GRADE)** |
|  | Assumed risk | Corresponding risk |  |  |  |
|  | **CD only** | **Bushenhuoxue soup with CD** |  |  |  |
| **Total effective rate** Follow-up: 3-13 months | **Study population** | | **RR 1.19**  (0.99 to 1.42) | 293 (3 studies) | ⊕⊕⊝⊝ **low**^1,2,3,4^ |
|  | **746 per 1000** | **888 per 1000** (739 to 1000) |  |  |  |
|  | **Moderate** | |  |  |  |
|  | **840 per 1000** | **1000 per 1000** (832 to 1000) |  |  |  |
| **HHS** Follow-up: 12 |  | The mean hhs in the intervention groups was **14.94 higher** (12.43 to 17.45 higher) |  | 120 (2 studies) | ⊕⊕⊝⊝ **low**^5,6^ |
| *The basis for the **assumed risk** (e.g. the median control group risk across studies) is provided in footnotes. The **corresponding risk** (and its 95% confidence interval) is based on the assumed risk in the comparison group and the **relative effect** of the intervention (and its 95% CI).  **CI:** Confidence interval; **RR:** Risk ratio; | | | | | |
| GRADE Working Group grades of evidence **High quality:** Further research is very unlikely to change our confidence in the estimate of effect.  **Moderate quality:** Further research is likely to have an important impact on our confidence in the estimate of effect and may change the estimate. **Low quality:** Further research is very likely to have an important impact on our confidence in the estimate of effect and is likely to change the estimate. **Very low quality:** We are very uncertain about the estimate. | | | | | |
| ^1^ All studies included exist high risk of bias ^2^ There exsit large heterogeneity across included studies ^3^ This outcome was evaluated according to subjective method ^4^ The total NO. of participants were 293 ^5^ All studies exsit large risk of bias ^6^ this outcome was evaluated using subjective method | | | | | |

**S7 Appendix**

| **Self-drafting Traditional Chinese Medical Prescription with CD versus CD** | | | | | |
| --- | --- | --- | --- | --- | --- |
| **Patient or population:** patients with osteonecrosis of femoral head **Settings:** inpatients **Intervention:** Self-drafting Traditional Chinese Medical Prescription with CD versus CD **Comparison:** CD only | | | | | |
| **Outcomes** | **Illustrative comparative risks* (95% CI)** | | **Relative effect (95% CI)** | **No of Participants (studies)** | **Quality of the evidence (GRADE)** |
|  | Assumed risk | Corresponding risk |  |  |  |
|  | **CD only** | **Self-drafting Traditional Chinese Medical Prescription with CD versus CD** |  |  |  |
| **Total effective rate** | **Study population** | | **RR 1.09**  (0.99 to 1.19) | 161 (2 studies) | ⊕⊕⊝⊝ **low**^1,2^ |
|  | **888 per 1000** | **967 per 1000** (879 to 1000) |  |  |  |
|  | **Moderate** | |  |  |  |
|  | **889 per 1000** | **969 per 1000** (880 to 1000) |  |  |  |
| *The basis for the **assumed risk** (e.g. the median control group risk across studies) is provided in footnotes. The **corresponding risk** (and its 95% confidence interval) is based on the assumed risk in the comparison group and the **relative effect** of the intervention (and its 95% CI).  **CI:** Confidence interval; **RR:** Risk ratio; | | | | | |
| GRADE Working Group grades of evidence **High quality:** Further research is very unlikely to change our confidence in the estimate of effect.  **Moderate quality:** Further research is likely to have an important impact on our confidence in the estimate of effect and may change the estimate. **Low quality:** Further research is very likely to have an important impact on our confidence in the estimate of effect and is likely to change the estimate. **Very low quality:** We are very uncertain about the estimate. | | | | | |
| ^1^ All studies included exist high risk of bias ^2^ This outcome was evaluated according to subjective method | | | | | |

**S8 Appendix PRISMA 2009 checklist**

| **Section/topic** | **#** | **Checklist item** | **Reported on page #** |
| --- | --- | --- | --- |
| **TITLE** | | |  |
| Title | 1 | Identify the report as a systematic review, meta-analysis, or both. | 1 |
| **ABSTRACT** | | |  |
| Structured summary | 2 | Provide a structured summary including, as applicable: background; objectives; data sources; study eligibility criteria, participants, and interventions; study appraisal and synthesis methods; results; limitations; conclusions and implications of key findings; systematic review registration number. | 2 |
| **INTRODUCTION** | | |  |
| Rationale | 3 | Describe the rationale for the review in the context of what is already known. | 3 |
| Objectives | 4 | Provide an explicit statement of questions being addressed with reference to participants, interventions, comparisons, outcomes, and study design (PICOS). | 3 |
| **METHODS** | | |  |
| Protocol and registration | 5 | Indicate if a review protocol exists, if and where it can be accessed (e.g., Web address), and, if available, provide registration information including registration number. | 3 |
| Eligibility criteria | 6 | Specify study characteristics (e.g., PICOS, length of follow-up) and report characteristics (e.g., years considered, language, publication status) used as criteria for eligibility, giving rationale. | 3-4 |
| Information sources | 7 | Describe all information sources (e.g., databases with dates of coverage, contact with study authors to identify additional studies) in the search and date last searched. | 4 |
| Search | 8 | Present full electronic search strategy for at least one database, including any limits used, such that it could be repeated. | 4 |
| Study selection | 9 | State the process for selecting studies (i.e., screening, eligibility, included in systematic review, and, if applicable, included in the meta-analysis). | 4 |
| Data collection process | 10 | Describe method of data extraction from reports (e.g., piloted forms, independently, in duplicate) and any processes for obtaining and confirming data from investigators. | 4-5 |
| Data items | 11 | List and define all variables for which data were sought (e.g., PICOS, funding sources) and any assumptions and simplifications made. | 4-5 |
| Risk of bias in individual studies | 12 | Describe methods used for assessing risk of bias of individual studies (including specification of whether this was done at the study or outcome level), and how this information is to be used in any data synthesis. | 5 |
| Summary measures | 13 | State the principal summary measures (e.g., risk ratio, difference in means). | 5 |
| Synthesis of results | 14 | Describe the methods of handling data and combining results of studies, if done, including measures of consistency (e.g., I^2^) for each meta-analysis. | 5 |
| Risk of bias across studies | 15 | Specify any assessment of risk of bias that may affect the cumulative evidence (e.g., publication bias, selective reporting within studies). | 5 |
| Additional analyses | 16 | Describe methods of additional analyses (e.g., sensitivity or subgroup analyses, meta-regression), if done, indicating which were pre-specified. | 5 |
| **RESULTS** | | |  |
| Study selection | 17 | Give numbers of studies screened, assessed for eligibility, and included in the review, with reasons for exclusions at each stage, ideally with a flow diagram. | 5-6 |
| Study characteristics | 18 | For each study, present characteristics for which data were extracted (e.g., study size, PICOS, follow-up period) and provide the citations. | 6-8 |
| Risk of bias within studies | 19 | Present data on risk of bias of each study and, if available, any outcome level assessment (see item 12). | 8 |
| Results of individual studies | 20 | For all outcomes considered (benefits or harms), present, for each study: (a) simple summary data for each intervention group (b) effect estimates and confidence intervals, ideally with a forest plot. | 8-12 |
| Synthesis of results | 21 | Present results of each meta-analysis done, including confidence intervals and measures of consistency. | 8-12 |
| Risk of bias across studies | 22 | Present results of any assessment of risk of bias across studies (see Item 15). | 5 |
| Additional analysis | 23 | Give results of additional analyses, if done (e.g., sensitivity or subgroup analyses, meta-regression [see Item 16]). | 12 |
| **DISCUSSION** | | |  |
| Summary of evidence | 24 | Summarize the main findings including the strength of evidence for each main outcome; consider their relevance to key groups (e.g., healthcare providers, users, and policy makers). | 12,13 |
| Limitations | 25 | Discuss limitations at study and outcome level (e.g., risk of bias), and at review-level (e.g., incomplete retrieval of identified research, reporting bias). | 13 |
| Conclusions | 26 | Provide a general interpretation of the results in the context of other evidence, and implications for future research. | 14 |
| **FUNDING** | | |  |
| Funding | 27 | Describe sources of funding for the systematic review and other support (e.g., supply of data); role of funders for the systematic review. | 14 |
